# Supplementary material for: Are carbon emissions trading and green financial instruments synergistic? -Comprehensive quantitative research based on content analysis
Source: PLoS One. 2024 Mar 7;19(3):e0298601. doi: 10.1371/journal.pone.0298601 (PMC10919601; doi:10.1371/journal.pone.0298601)
Supplement: S1 Data — (ZIP) [file pone.0298601.s001.zip › Data/地级市Z_F工作报告.zip!/║┌┴·╜¡/┤≤╟∞╩╨/└┤╫╘╣1⁄2╓┌║┼í╛┬φ┐╦╩2╛▌═°í┐_2019.docx]

各位代表：

　　现在，我代表市人民政府向大会报告工作，请予审议，并请市政协委员提出意见。

　　一、2018年工作回顾

　　过去的一年，我们高举习近平新时代中国特色社会主义思想伟大旗帜，全面贯彻落实党的十九大精神，深入贯彻落实习近平总书记对黑龙江省两次重要讲话，以及在深入推进东北振兴座谈会上的重要讲话和考察黑龙江的重要指示精神，坚决贯彻落实省委、省政府各项决策部署，在市委的坚强领导下，围绕“争当排头兵、建设新大庆、推动全面振兴全方位振兴”的奋斗目标，弹好“四重奏”、勇闯“四条路”，努力推动经济社会持续健康发展，较好地完成了十届人大三次会议确定的各项目标任务。

　　——城市实力得到新提升。全市经济运行速度稳步回升、质量稳步提高。预计实现地区生产总值2800亿元、增长了3.5%，人均GDP10.3万元、同比增加了0.5万元，扣除中直部分地方经济增长了7.3%，非油经济、非公经济分别实现了6.8%和9%的中高速增长。全市地方财政总收入完成385.2亿元、增长了2.5%。农业稳产增效，增加值增长5.8%。粮食总产量实现了82.4亿斤，畜牧业增加值增长了5.3%。工业加速回暖，增加值增长1.3%。大庆油田完成油气当量4166.8万吨，“压舱石”地位依然坚实。地方规上工业增加值增长了14%。服务业贡献率进一步提升，增加值增长7.2%，拉动GDP增长2.4个百分点。社会消费品零售总额实现1240亿元、增长了7%，位居全省前列。商贸骨干企业带动批发和零售业实现增加值306亿元、增长了16.6%。大庆市位列2018年中国百强城市排行榜第68位、中国产业竞争力城市百强榜第87位。

　　——重点产业取得新突破。“雁阵式”产业板块基础更加夯实，石化公司炼油结构调整转型升级项目全面启动，炼化公司30万吨烷基化等7个项目建成投产，550万吨重油催化热裂解项目正在加快推进，百万吨下游加工原料基本得到落实。沃尔沃大庆工厂年产轿车5.2万辆，S60新车型正式落地，汽车产业骨架已经形成。华为云计算数据中心上线运行，伊品玉米深加工一期工程试车生产，宏福现代农业小镇一期植物工厂建成投产，豫港龙泉铝材加工项目开工建设，大庆汽车城项目全面运营，国家可再生能源综合应用示范区成功获批。

　　——动能转换迈出新步伐。投资拉动强劲，全市落实投资千万元以上产业项目230个，其中亿元以上109个。固定资产投资完成472亿元、增长了7%，其中产业项目投资380亿元、占固定资产投资比重达到了80.5%。创新驱动效果显现，中科创业园成为国家级孵化器，东北石油大学“原油整形装置”项目荣获中国专利金奖。全市新增高新技术企业37家，高新技术产业增加值突破了800亿元。市场活力持续增强，新增市场主体4.1万户，全市11个电商园区入驻互联网企业325家，电商交易额达到105亿元、增长了16.6%。全市接待游客2506万人次、增长了35.7%，大庆获得中国工业旅游创新成果奖。消费金融、汽车金融等业态加快发展，金融业增加值增长了7.4%。招商引资扎实推进，“名企入庆、定向招商、暖企助力”行动成效明显，全年招来亿元以上项目130个，实际到位资金增长了9.3%。

　　——城乡面貌呈现新变化。乡村振兴走在全省前列，新建改建农村饮水安全工程283处、农村公路348.9公里，改造农村危房11865户、改厕1719户，完成工程造林4.2万亩，农村生产生活条件大幅改善。淘汰燃煤小锅炉11台、实现并网47万平方米，改造城区道路17条、供热供水供气管网104公里，建设视频监控系统6330套，城市公共设施不断完善。三永湖、万宝湖水体治理达到销项标准，集中式饮用水水源地专项整治提前完成。空气质量优良天数达到了340天，创2012年新空气标准实施以来最好水平，中央环保督察“回头看”移交的信访问题得到了有效解决。

　　——改革开放带来新活力。农村土地确权改革和集体产权清产核资全面完成，“三权分置”的格局正在形成。驻庆央企“三供一业”及办社会职能分离移交稳步推进。市城投公司、产业服务公司等国企整合转隶基本完成，市热力集团、水务集团营业收入较改革前增长了15%。开放合作深度推进，中俄原油管道二线工程投入运营，管输俄油实现了2830万吨。高质量承办了第十四届省运会、第七届省残运会、斯诺克国锦赛等13项赛事。机场旅客吞吐量突破了83万人次、增长了33.7%。外贸进出口总值达到990亿元、增长了73%，总量和增速均列全省首位。

　　——营商环境有了新改善。以“只进一扇门”、“最多跑一次”为重点的“放管服”改革成效显著，审批、服务事项大幅压缩，一些事项实现了网上可办、就近能办、同城通办。企业投资项目承诺制在我市高新区、经开区落地实施，政府出资新组建的产业基金，有力地支持了重大项目建设。中小微企业发展的优惠政策得到落实，诚信体系建设不断加强，我市信用综合指数在全国262个地级城市中排名第44位。

　　——民生事业实现新进步。全市民生支出235.1亿元，占公共财政支出比重达到了78.2%。城乡居民人均可支配收入分别达到了40092元、15731元，增长了4%和6.6%。城镇新增加就业人数同比提高了8%，城镇登记失业率3.76%、同比下降了0.4个百分点。脱贫攻坚取得重要进展，杜尔伯特县摘掉省级贫困县帽子，全市共有31个贫困村退出了贫困序列，有3279户6996人摆脱了贫困，282个产业扶贫项目得到落实并发挥了积极作用。教育事业均衡发展，全市有8所幼儿园晋升为省级示范园。市民关注的中小学“阳光分班”在市区顺利实施，6.5万名新生受益。医疗卫生事业加快发展，公立医院改革持续深化，药品购销“两票制”顺利实施。全市共建成了医联体和医共体28个、危重孕产妇和新生儿救治中心10个，组建了家庭医生团队626个。公共文化活动丰富多彩，国家公共文化服务体系示范区启动建设，“大庆之冬”、“激情之夏”等文化活动深入人心。社会保障水平切实提高，城乡居民医保制度在省内率先实现了“八统一”，低保标准实现了“十四连增”，城乡居民基础养老金人均增资20%。社会治理体系持续健全，公安科技大厦投入使用，“扫黑除恶”专项斗争纵深推进，八类主要刑事案件发案率创2000年以来最低。重点领域安全整治保持高压态势，全年未发生重特大安全生产事故。社会矛盾得到有效化解，建立并完善了律师介入信访工作机制，人民调解“4543”大庆模式被誉为新时代“枫桥经验”并推向全国。民兵调整改革专项工作在中央军委评比检查中位居全国前列。

　　——政务建设见到新成效。中央八项规定及实施细则精神、省委省政府和市委的各项规章制度，得到了严格执行。机关作风逐步转变，“一岗双责”深入落实，党风廉政建设不断强化，政府自身建设进一步加强，政务运行效率效能明显提高。政府自觉接受市人大法律监督、工作监督和市政协民主监督，依法监督意识进一步增强。267件人大代表建议和251件政协委员提案全部办结。主动接受市监察委依法监察，确保了公权力规范运行。

　　气象、统计、审计、双拥、国防动员、外事侨务、民族宗教等工作，以及残疾人、妇女儿童等事业都取得了长足进步。

　　各位代表!

　　过去的一年，大庆市经济社会发展取得的成绩来之不易，这是省委省政府和市委坚强领导的结果，是市人大、市政协监督支持的结果，是油化央企、驻庆部队、中省直单位、高校院所和社会各界共同努力的结果，更是全市人民团结一心、艰苦奋斗的结果。在此，我代表市人民政府，向各位代表、各位委员和全市人民，向所有关心支持大庆发展的同志们、朋友们，表示崇高的敬意和衷心的感谢!

　　在看到成绩的同时，我们也要清醒地看到存在的差距和不足。我市经济总量相对较小、经济运行质量不高，经济发展速度低于全省平均水平;民营经济偏弱，县域经济不强，农村基础设施和公共服务欠账较多，营商环境需要进一步优化;部分公务人员思想观念陈旧、干事劲头不足、专业能力欠缺，不担当不作为现象依然存在。这些问题需要我们在今后的工作中高度重视，并认真加以解决。

　　二、2019年工作安排

　　2019年，是新中国成立70周年、大庆油田发现60周年和建市40周年，是我们决胜全面建成小康社会的关键之年，更是大庆转型振兴再出发的奋进之年，做好今年的政府工作责任重大。

　　新的一年，我们要抢抓机遇、乘势而上。习近平总书记勉励我们“大庆就是全国的标杆和旗帜”，“要以‘油头化尾’为抓手，推动石油精深加工，推动‘油城’发展转型”。省委要求大庆“大力弘扬大庆精神铁人精神，争当全国资源型城市转型发展排头兵”。这为推动大庆转型振兴注入了强劲的动力，我们必须牢牢把握发展机遇，在东北振兴发展的大格局中找准定位、抢抓先机、加快发展。

　　新的一年，我们要勇于担当、奋力前行。市委九届三次全会遵循发展新航向、贯彻振兴新要求、把握市情新特征、立足阶段新定位，对当前和今后一个时期大庆全面振兴全方位振兴的战略思路、目标任务作出了整体部署。市委九届四次全会就深入贯彻落实中央和省委经济工作会议精神，推动大庆争当“排头兵”的阶段性任务作出了再安排再部署。我们要在市委的坚强领导下，保持战略定力，坚持不懈、艰苦奋斗，切实抓好各项任务的贯彻落实。

　　新的一年，我们要放大优势、深挖潜力。大庆坚实的工业基础积累了雄厚产业优势，接续产业项目集群建设形成了投资吸引优势，市政设施、城市功能、科研教育等构成了综合承载优势，大庆精神铁人精神涵养了独特人文优势。我们必须发挥好这些比较优势，努力在东北全面振兴全方位振兴的时代进程中抢占高地，在全国资源型城市转型发展中抢站排头。

　　新的一年，我们要直面挑战、扎实工作。受国际经济形势影响，加之油价波动频繁，叠加油田产量战略调减等因素，我市经济发展面临诸多困难。我们必须迎难而上、知难而进，拿出“有条件要上，没有条件创造条件也要上”的勇气，采取积极的应对措施和务实有效的招法，全力开创各项工作的新局面。

　　2019年，政府工作总的要求是：以习近平新时代中国特色社会主义思想为指导，聚焦贯彻落实习近平总书记在深入推进东北振兴座谈会上的重要讲话和考察黑龙江的重要指示精神，统筹推进“五位一体”总体布局、协调推进“四个全面”战略布局，践行新发展理念，落实高质量发展要求，坚持稳中求进工作总基调，坚持以供给侧结构性改革为主线，围绕中央经济工作会议强调的“六稳”要求、“八字”方针，深入落实省委省政府各项决策部署和市委九届三次、四次全会思路目标，全力以赴抓发展、促改革、优环境、惠民生、转作风，奋力推动大庆全面振兴全方位振兴，加快争当全国资源型城市转型发展排头兵。2019年的主要预期目标是：地区生产总值增长4%，地方经济增长7.5%，规上工业增长1.3%，制造业占GDP比重提高到15%，万元GDP能耗下降3.2%。公共财政预算收入增长2.9%，固定资产投资增长8%，社会消费品零售总额增长6%。城乡居民人均可支配收入分别增长4%和6%。城镇登记失业率控制在4.3%以内。

　　完成好今年各项工作目标，不仅要解放思想、勇于担当，更要脚踏实地、真抓实干，在高标准推动日常工作的同时，对标市委争当“排头兵”2019年行动计划，重点要抓好10个方面50项具体工作任务：

　　(一)抓共建促融合，巩固油化经济根基。大庆因油而生、因油而兴。我们要坚持“四共方针”，落实好地企协调会议机制，在对上争取、产业培育、项目建设、科技创新、园区共建、城市管理、社会治理等方面加大融合发展力度。(1)要稳定油气能源生产。全力以赴支持油田公司打好勘探开发、科技进步、降本增效等“抢产夺油”攻坚战，确保大庆油田油气当量稳定在4000万吨以上，巩固经济发展的“压舱石”地位。(2)要稳步发展中直化工。支持石化公司炼油结构调整转型升级项目建设，力争连续重整装置子项目9月份建成投产，夯实“油头”基础。支持炼化公司石蜡项目建设，丰富下游产品结构。支持中蓝石化公司实施技术改造升级项目，提升炼油加工能力。(3)要合力推动改革创新。扎实推进驻庆央企“三供一业”及办社会职能分离移交，启动物业、供热等设施维修改造工程。共建国家级可再生能源综合应用示范区，抓好大庆油田和经开区两个国家级增量配电业务改革试点。(4)要大力深化科技融合。建立油化央企、地方政府和驻庆高校科技资源三方共用共享机制，培育一批科技成果产业化的平台载体。加快突破体制机制限制，以油田公司为主，融合东北石油大学，建设国家能源陆相砂岩开采研发中心，打造全国领先的科技创新平台。

　　(二)抓投资上项目，积蓄转型发展潜能。2019年，我们要围绕打造十大“雁阵式”产业板块，新建续建千万元以上产业项目230个，其中超亿元项目120个、计划完成年度投资310亿元。(5)要全力推动项目前期准备。各级政府和市直部门要树立项目建设“全年无淡季”的思想，超前准备、提早动手，在方案设计、立项审批、手续办理、资金筹措和招标采购等环节提供全方位的支持，确保项目落地开工准备最快、用时最少、效率最高。今年将重点开工建设星云化工70万吨聚碳酸酯、海润20万吨醋酸制乙醇等“化尾”项目，为承接好石化公司新增化工原料做好准备;开工建设宏福现代农业二期、金锣大豆蛋白加工等项目，为提升农牧产品加工转化能力打下基础;开工建设溢泰240万片砷化镓晶片等项目，建成投用化合物半导体产业园;开工建设数字高新智慧园区、万达主体街区改造、中移动生产调度中心等项目，带动服务业升级。(6)要全力推动项目包保服务。制定实施首席服务官代办制度、服务团队驻场制度，为纳入全省产业项目三年行动计划和全省“百大”项目建设计划的项目，提供“一对一”的个性化服务。重点支持550万吨重油催化热裂解项目加快建设，力争9月份部分中交;支持沃尔沃扩能改造，确保S60样车4月份下线、11月份量产;启动运营大庆汽车城三期，建设汽车文化公园;支持豫港龙泉铝材加工项目续建，完成一期精深加工部分的18栋厂房及配套工程;支持伊品玉米深加工二期、国投30万吨燃料乙醇等项目建成投产，拉长“粮头食尾”、“农头工尾”等精深加工链条。(7)要全力推动项目招商引资。建立“专班+园区”、“目标+考核”的招商机制，紧盯长三角等重点区域承接产业转移开展定点招商，紧盯重点企业做好供应端、采购端、研发端项目的配套招商，紧盯油化装备市场和驻庆化工央企的原料供给、产品市场进行招商，全年力争引进亿元以上项目120个。(8)要全力推动项目谋划储备。加快重点产业项目谋划，建立市级重点产业项目库，对入库项目实施台账管理、动态监测、落地考核。坚持解放思想谋项目、抢抓政策机遇谋项目，加紧谋划一批油化装备制造存量资源裂变的项目、龙头企业上下游关联集聚的项目、新业态新经济新平台催生的项目，积攒我市产业发展后劲。

　　(三)抓存量引增量，提升地方工业质量。打通要素配置路径、企业成长路径、产业育成路径，推动地方工业高质量发展。(9)实施保规晋规计划。坚持“抓大不放小”，采取措施分类推进停产半停产企业释放有效产能，引导“潜力股”企业扩能改造，支持临规企业加强技术储备、改进工艺路线、优化产品结构，力争新增规上企业46户。(10)推动既有存量裂变。制定存量资源盘活专项支持政策，通过招商盘活一批资源闲置、产权关系明晰的企业;采取股权融资、资产质押、债转股等方式扶持一批资金短缺、资金链断裂、经营困难的企业;启动司法拍卖程序清理一批严重违约、资不抵债、圈占土地的企业;推动成果转化落地一批实验室研发与产业化应用有效嫁接孵化的项目，实现存量资源转换升级。(11)提升园区基础功能。引进专业管理机构和团队，市场化运营经开区科技企业孵化器。启动建设石化产品加工产业园、新型功能材料产业园和天然气专业园区，加快推进林源热电站、宏伟污水处理厂等园区配套项目，不断提升园区产业承载能力。支持德力戈尔工业园区申报省级开发区。

　　(四)抓振兴补短板，加快农业农村发展。扎实推进乡村振兴战略，抓好农业供给侧结构性改革，持续增强“三农”发展内生动力。(12)全面提升种植业。落实粮食安全市长责任制，推动粮食生产由增产向提质转变。依托大庆国家现代农业示范区，建设农业“三减”示范基地200万亩、绿色食品生产基地240万亩，种植优质玉米600万亩、水稻200万亩、大豆100万亩，粮食总产稳定在100亿斤以上，给放心安全的“中国饭碗”多装“大庆粮食”。(13)加快发展畜牧业。要统筹生态可承载、环境可持续，科学规划畜牧养殖，坚持“养加”一体化发展。推进中以奶业、兴和10万吨蛋品、子赫牧业120万羽大鹅、乐尔3万只种兔、大北农生猪等项目建设。建好畜牧防疫队伍，加大技防投入力度，强化畜禽疫病防控。(14)差异化发展县区经济。强化顶层设计，出台支持县区经济发展的具体政策措施。建立市财政统筹的税收分成机制，鼓励在全市省级以上园区间发展“飞地经济”。围绕“一县一品、一县多品”，立足多领域全覆盖，支持各县区培育1-2个特色优势产业，抓好2-3个立县立区产业项目。(15)全力打赢脱贫攻坚战。深入实施脱贫攻坚三年行动计划，持续推进产业、就业、金融、健康等综合扶贫措施，确保林甸县脱贫摘帽，全市剩余的10个贫困村全部出列，杜尔伯特县、肇州县、肇源县和大同区贫困人口全部销号。新建续建产业扶贫项目42个，增强“造血”能力。支持林甸伊利乳业项目建设，打造高端乳制品生产中心。(16)促进农村生产要素优化配置。深化农村集体产权制度改革，在486个村级单位组建股份经济合作社，提升村集体经济实力。

　　(五)抓供给带需求，推动消费市场升级。立足扩大有效消费，进一步提升供给能力、改善消费预期、激发消费潜力。(17)推动“文化+旅游”融合发展。抓好国家公共文化服务体系示范区创建，完善市、县、乡、村四级公共文化设施，确保通过中期督查。大力推进大庆旅游全产业链一体化项目，精心策划石油工业游、湿地温泉游、草原自驾游、乡村休闲游，办好中国(大庆)湿地旅游文化节等活动，加快打造中国北方四季风情全域旅游示范城市。(18)推动“竞技体育+群众体育”并进发展。强化与国际国内体育赛事协会组织的广泛合作，办好斯诺克国锦赛、全国铁人三项联赛和全国田径锦标赛等专业性赛事。大力开展“青少年上冰雪”活动，高水平承办全国帆船帆板俱乐部分站赛、黑龙江省第六届体育舞蹈锦标赛等群众性赛事。(19)推动“线下实体+线上平台”互动发展。推进肇源县、林甸县国家电子商务进农村示范县建设，支持线下商贸企业、本土电商企业和国内知名电商平台开展战略合作，扩大惠丰乳品、老街基食品等特色产品网络销售，力争电子商务交易额增长10%。(20)推动“健康+养老”组合发展。建立健全养老机构综合评价体系。放大寒地冰雪、特色温泉、绿色湿地等生态优势，推动既有旅游项目植入养老功能、提供养老服务。利用厂矿医院、社区照料中心等资源优势，开发微型养老、就近养老、康复养老产品。利用社区卫生机构、数据运维平台等优势，探索建立社区居家养老电子健康档案系统，推进智慧养老、数字养老等业态加快发展。

　　(六)抓服务提效能，系统优化营商环境。围绕打造东北地区营商环境最优城市，进一步创新各项便企便民举措。(21)支持民营经济加快发展。推动民营经济发展33条政策措施落地生效，构建“个转企、小升规、规改股、股上市”梯次培育机制，对贡献突出、成长性好的民营企业，给予贷款贴息、产业基金引导等“一企一策”专项扶持。(22)加快推行“1+4”政务服务。建立“一网”纵向联通省、市、县区、乡镇和街道，横向联通专业部门的“四级网络”，实施跑一次、不见面、延伸办理、复杂事项“四个清单”管理，建好实体、网络、移动、自助“四种形式”的服务大厅，确保90%以上事项实现“最多跑一次”，70%以上事项实现“网上办”。动态梳理行政权力事项清单和工作流程，能取消的坚决取消，能下放的全部下放。推进证照分离改革，完善市场主体登记全程电子化系统，为市场主体创新创业提供便利。(23)完善“一件事”联审联办机制。对固定资产投资事项实行“一表申请、一窗受理、疑难会商、限时办结”。进一步压缩商事登记、不动产登记等即办事项办理时限，企业设立时间控制在8天之内。(24)提高公共资源配置效率。建设公共资源交易远程开标系统、政府采购网上电子商城，实现政府采购、建设工程、交通水利等领域交易活动全程网上运行。(25)拓宽企业融资渠道。制定政府出资产业基金管理办法，更好发挥财政资金撬动作用。支持银行机构开展供应链金融、知识产权质押等产品创新，通过推动混改和引入国开行等中长期优惠资金，更好服务国企债务融资和项目建设融资，扶持同禹药包公司等重点企业上市。打好防范化解重大风险攻坚战，有效化解政府债务，守住不发生区域性系统性金融风险底线。(26)加大人才引育力度。落实高端人才引进“头雁行动”，加快引进一批企业家人才，更好服务“雁阵式”产业板块培育和市属国企改革。实施“人才引进高校行”、“双百培养计划”等人才工程，帮助高校院所、中小学校和医疗机构引进专业技术和管理人才，帮助地方企业引进技术人员和产业工人，用更加灵活、务实、管用的政策措施引人留人育人。(27)强化科技创新。实施新一轮科技型企业三年行动计划，推进高新区双兴智能科创中心等平台建设，培育重点实验室2个，转化科技成果150项以上。(28)构建亲清新型政商关系。制定出台构建亲清新型政商关系的意见，厘清政商界限，明确服务任务，提高服务质量。完善信息公示和联合惩戒机制，推进政务诚信、商务诚信、社会诚信和司法公信建设。健全受理损害营商环境行为的投诉举报制度，依法打击破坏营商环境的行为。

　　(七)抓改革扩开放，激发微观主体活力。突出关键领域和环节，促进域内域外要素优化配置。(29)推进重点改革。深化政府机构改革，清晰界定部门职能。深化高新区、经开区管理体制改革，探索赋予决策、审批、人事、运营及社会管理等方面的市级权限。深化建筑工程领域管理机制改革，全面落实政府投资项目“双主体”责任，建立工程款按合同拨付、农民工工资按月支付的管理和监督办法。深化市场化改革，推动政府非办公类资产和企业非经营性资产向创造财富的生产部门配置。深化市属国资国企改革，组建国有资本投资运营公司，抓好市外贸公司等3户困难企业处置出清，撤并23家长期无经营活动、经营性亏损的市属企业二三级子公司;完善市属国有企业经营业绩考核机制，对减亏、增盈、减少财政补贴的给予一定比例奖励;完善国有企业内部“三项制度”，尽快形成管理人员能上能下、职工能进能出、收入能增能减的机制，建立健全与劳动力市场基本适应、与企业经营效益和劳动生产率挂钩的工资决定和正常增长机制。(30)深化市校院所共建。创造条件建立中国地质调查局、大庆油田、大庆市政府和相关科研院所之间的合作机制，在地热水回灌技术、废弃油井综合利用、水资源环境等方面，开展技术攻关和产业化实践及应用。支持八一农垦大学、省科学院大庆分院等高校院所，在以色列奶牛疾病防治技术本地化、汉麻精深加工等领域，加快产学研用协同创新。鼓励高校围绕地方产业发展和企业需求，科学设置专业，为沃尔沃、豫港龙泉等龙头企业定向培养人才。(31)加强庆惠合作。建立大庆与惠州政府间定期会商机制，研究重大事项、协调重大问题。支持高新区、经开区与惠州的产业园区精准对接，在项目承接、技术嫁接、供需衔接上再落地一批新成果。(32)扩大开放交流。加快建设海关监管作业场所和B型保税物流中心。推动大庆与美国休斯敦、韩国昌原缔结国际友好城市，加入世界冬季城市市长会议组织，与世界能源城市伙伴组织开展务实合作，加快大庆对外开放的步伐。

　　(八)抓治理强修复，保护绿色生态环境。坚持“两山论”理念，下力气保护好大庆生态环境，全力构建“市县乡村企联动、水林田湖草齐抓”的工作格局。(33)开展全域全民绿化。通过政府引导、地企共建、市场运作、居民认领，推动空地插绿、公园增绿、围矿添绿、绕湖补绿、护村植绿、环城扩绿，推进油田核心区生态修复，打造一批公益绿地、纪念林廊和彩化景观。(34)加强污染综合防治。严格落实河湖长制，抓好“清四乱”专项行动，重点解决好松花江、嫩江等水域及沿岸的乱占、乱采、乱堆、乱建问题。强化明湖、燕都湖和东风湖综合治理，完成大龙虎泡水源地替代工程建设。持续打好蓝天保卫战，逐步淘汰县域建成区燃煤小锅炉，全域禁止秸秆露天焚烧。(35)推动城乡垃圾治理。编制城乡垃圾综合治理专项规划，探索推进以乡镇转运站、村屯收集站为重点的农村生活垃圾收运体系建设，抓好肇源县生活垃圾资源化利用省级试点。加快实施大庆市生活垃圾综合处理厂焚烧发电技改项目，开工建设大庆市餐厨废弃物处理厂，加快运营东城区第二污水处理厂，启用大庆市建筑垃圾消纳场，制定实施垃圾收费管理办法，系统推进城市“五类垃圾”的分类处理。

　　(九)抓管理重精细，提高城市建管水平。加大城市“双修”力度。(36)打造顺畅交通环境。健全城市交通导向系统，集中完善城市干道出入口标识牌、夜间可视化指示牌。根据季节因素动态调整公交车班次，投放新能源公交车100辆，方便市民出行。加强停车管理，坚决整治停车乱象。(37)补齐城市重大功能短板。升级萨尔图机场功能，开工建设国际航站楼，启动航空口岸开放申请工作。推动绥沈公路肇松段、哈肇高速公路大庆段、哈大与大齐高速公路连接线等项目建设，打通大庆对外交通瓶颈。实施经七街和纬十二路延伸改造项目。新建西城客运综合枢纽，打造集铁路、公路、公交等多种运输方式于一体的换乘中心。(38)创新网格管理模式。出台城市精细化管理实施办法，明确管理标准和责任体系。赋予街道层级深度参与城市管理的权限，建立“居民需要、街道呼叫、部门响应、企业即到”的服务模式。(39)综合整治市容环境。建立路灯、井盖、公厕等“城市家具”档案，推进城市标识配套、街路硬化铺装、城市亮化改造。全面排查治理城市环境脏乱差、基础设施丢失破损、违规违法私搭乱建、庭院绿地小开荒等突出问题，提升城市品位。(40)推进城市细部治理。逐年分步实施油井用地、废弃地、边角地改造，建设完善口袋公园、微型广场。提高铁路东西两站、萨尔图机场等城市窗口部位综合管理和服务水平。加强物业监管，规范物业服务项目，下力气整治物业管理不到位、服务不达标等突出问题，建立群众认可、业主评判的物业企业考核新机制，实施物业服务行业优胜劣汰。规划建设一批便民小市场，为解决低收入群体就业、方便社区居民生活创造必要条件。

　　(十)抓实事用真情，更好保障改善民生。重点实施“十项暖心行动”：(41)大力度实施就业促进行动。搭建驻庆高校毕业生本地就业服务平台，让更多的大学毕业生留在大庆就业创业。抓好自主就业退役士兵职业教育，阳光安置符合条件的退役士兵。强化职业技能培训，扶助农民工、就业困难人员等重点群体就业。(42)大力度实施教育提质行动。推动学前教育、义务教育、高中教育、职业教育均衡发展，扩建兰德学校，推进米兰小镇二期幼儿园尽快投入使用。推动教育资源优化配置，实施高中教学质量提升计划，扩大职业教育“双元制”改革试点。出台深化新时代教师队伍建设改革的意见。(43)大力度实施全民健康行动。深化医疗体制改革，探索实行公立医院薪酬制试点和市场化的智慧健康服务试点。为社区卫生服务中心招聘专业技术人员，为乡镇卫生院动态补充医学院校毕业生。建设农民体育健身工程，实现行政村公共体育健身设施全覆盖。(44)大力度实施文化惠民行动。开展社会主义核心价值观和大庆精神新时代内涵“七进”教育，推动文明单位、文明家庭、文明校园等基础创建常态化。加强文化精品创作，推出音乐剧《铁人》等一批歌颂祖国、歌颂党、歌颂大庆的文艺作品。(45)大力度实施社保提标行动。建设“12+1+N”特色社会救助体系，进一步提高城乡低保和特困人群供养标准。推进机关事业单位养老保险制度改革，落实困难职工基本养老保险助保贷款政策。(46)大力度实施情暖大庆行动。落实特殊困难群体关爱救助机制，建设爱心驿站、公益超市，关注关爱农村“三留守”人员、残障群体和鳏寡孤独老人。(47)大力度实施老区改造行动。通过住房专项维修资金调剂、争取上级政策补贴等方式，推进悦民苑、利民苑等老旧小区的供热供水、居住环境和楼体外立面改造。(48)大力度实施冬日暖居行动。建立低温小区、低温楼、低温户台账，统筹热源能力，优化热网布局，实施现有老旧供热管网分期分批改造工程，逐个供热企业确定年度改造计划。(49)大力度实施平安创建行动。强化应急体系建设，提升应急处置能力。落实安全生产责任制，下力气抓好矿区、危爆物品、道路交通、建设工程、消防等领域专项整治，坚决遏制重特大安全事故发生。深化智慧警务建设，加强警民融合、警企共建，持续开展“扫黑除恶”专项斗争，探索构建防控前置、高效打击的社会治安防控新格局。(50)大力度实施基层治理行动。探索推动社会管理模式创新，充分运用律师、人民调解介入信访工作的“大庆模式”，依法处理各类信访问题和信访积案，最大限度把矛盾化解在初始状态，确保社会和谐稳定。

　　各位代表!

　　完成2019年经济社会发展目标，任务艰巨，时不我待，唯有苦干实干，才能善作善成。

　　一要坚持党的领导。切实增强“四个意识”，坚定“四个自信”，做到“两个坚决维护”。要持续强化理论武装。深刻领悟习近平新时代中国特色社会主义思想、党的十九大精神，以及习近平总书记重要讲话和重要指示精神，不断推动新思想新理念新要求往深里学、往心里走、往实里做。要持续强化政治担当。推进“两学一做”学习教育常态化制度化，认真开展“不忘初心、牢记使命”主题教育，领导干部带头讲规矩、讲程序、讲奉献，带动全员敢较真、敢碰硬、敢作为。要持续强化行动自觉。在市委坚强领导下，紧紧围绕“争当排头兵、建设新大庆”的战略安排、方向路径及重点任务，找准政府各项工作发力点，逐一对标承接，逐一细化载体，逐一压实责任。

　　二要坚持解放思想。深入开展解放思想推动高质量发展大讨论。要在深化市情认识上解放思想。既要夯实油经济又要加速发展非油经济，既要完善城市基础功能又要同步释放综合承载能力，既要干好当前又要谋划长远。要在转型发展上解放思想。紧紧围绕克服资源精深加工链条之短、民营经济活力不足之短、科技成果转化利用之短、自然生态环境欠账之短、城乡精细管理缺位之短，想破解之法、求转型之道。要在攻坚克难上解放思想。全面提高驾驭复杂矛盾问题的能力，在应对各种风险挑战上开创性地“试”，在破除体制机制障碍上创造性地“做”，在推动重点工作落实上突破性地“抓”，力求定下的事一抓到底、见到成效。

　　三要坚持狠抓落实。以钉钉子的精神，全面深入贯彻中央、省委和市委各项重大战略、方针、政策，确保不打折扣、落实到位。要保持连续性抓落实。强化政府机构调整后的职能对接，各部门的班子要尽快进入角色，队伍要尽快进入状态，工作要尽快提升水平。要保持衔接性抓落实。健全政府运行机制，完善实施周例会、月报告、季考核，以及政府班子和各委办局“AB”岗制度，形成无缝抓落实的工作链条。要保持时效性抓落实。树立结果导向思维，建立量化工作图谱，倒推任务目标、倒排时间节点。同时，专人专班全过程督办，确保层层有限定之责，事事有专管之人，时时有反馈之声。

　　四要坚持依法行政。自觉运用法治思维和法治方式推动工作，确保各项行政行为始终在法治轨道运行。要做到敬畏法律。坚持政府法律顾问、常务会议会前集体学法等制度，健全重大决策合法性审查、风险评估和责任追究机制，切实做到法定职责必须为、法无授权不可为。要做到公正透明。完善政府信息公开目录，推进政府决策、执行、管理、服务、结果“五公开”。加强行政复议和行政应诉，依法依规处置历史遗留问题。要做到渠道畅通。政府公务人员要以换位思考的方式，及时回应群众诉求，建立市长热线、网络问政“快转速办、快答实办”机制。要做到主动接受监督。自觉接受市人大及其常委会的法律监督、市政协的民主监督和市监察委依法监察，广泛接受社会监督和舆论监督。

　　五要坚持勤政廉政。从市政府一班人做起，带动全市政府系统多想事、多干事，干净干事、干事干净。要保持良好工作状态。发现问题不回避、遇到矛盾不躲闪、面对压力不退缩。要深入基层搞好调查研究。要勤到县区、勤入园区、勤访社区。要树立过紧日子思想。坚持尽力而为、量力而行，注重勤俭节约，把财政有限的资金用在刀刃上。要严守纪律和规矩底线。严格落实中央八项规定及实施细则精神，严格执行《准则》和《条例》，认真落实党风廉政建设主体责任和“一岗双责”，认真执行市委第一种形态实施办法，深入查摆廉政风险，强化警示教育，规范权力运行，确保干部清正、政府清廉、政治清明。

　　各位代表!

　　回首过去，我们克服了一个又一个困难，取得了一个又一个进步!面向未来，我们满怀信心、斗志昂扬!让我们在市委的坚强领导下，大力传承和弘扬大庆精神铁人精神，进一步解放思想、改革创新，进一步凝心聚力、攻坚克难，进一步干在实处、走在前列，为全面建成小康社会，为推动大庆全面振兴全方位振兴，为大庆争当全国资源型城市转型发展排头兵作出积极贡献!
